# Sugar-Lowering Drugs for Type 2 Diabetes Mellitus and Metabolic Syndrome—Review of Classical and New Compounds: Part-I

**DOI:** 10.3390/ph12040152

**Published:** 2019-10-10

**Authors:** Raquel Vieira, Selma B. Souto, Elena Sánchez-López, Ana López Machado, Patricia Severino, Sajan Jose, Antonello Santini, Ana Fortuna, Maria Luisa García, Amelia M. Silva, Eliana B. Souto

**Affiliations:** 1 Department of Pharmaceutical Technology, Faculty of Pharmacy, University of Coimbra (FFUC), Pólo das Ciências da Saúde, 3000-548 Coimbra, Portugal; raquelvieira.fmuc@gmail.com; 2 Department of Endocrinology, Hospital São João, Prof. Alameda Hernâni Monteiro, 4200-319 Porto, Portugal; sbsouto.md@gmail.com; 3 Department of Pharmacy, Pharmaceutical Technology and Physical Chemistry, Faculty of Pharmacy and Food Sciences, University of Barcelona, Institute of Nanoscience and Nanotechnology (IN2UB), 08028 Barcelona, Spain; esanchezlopez@ub.edu (E.S.-L.); marisagarcia@ub.edu (M.L.G.); 4 Centro de Investigación Biomédica en Red de Enfermedades Neurodegenerativas (CIBERNED), University of Barcelona, 08028 Barcelona, Spain; lora_ana@hotmail.com; 5 Laboratory of Nanotechnology and Nanomedicine (LNMED), Institute of Technology and Research (ITP), Av. Murilo Dantas, 300, Aracaju 49010-390, Brazil; pattypharma@gmail.com; 6 University of Tiradentes (UNIT), Industrial Biotechnology Program, Av. Murilo Dantas 300, Aracaju 49032-490, Brazil; 7 Department of Pharmaceutical Sciences, Mahatma Gandhi University, Cheruvandoor Campus, Ettumanoor, Kerala 686631, India; sajanjose@hotmail.com; 8 Department of Pharmacy, University of Naples Federico II, Via Domenico Montesano, 49-80131 Naples, Italy; antonello.santini@unina.it; 9 Department of Pharmacology, Faculty of Pharmacy, University of Coimbra (FFUC), Pólo das Ciências da Saúde, 3000-548 Coimbra, Portugal; anacfortuna@gmail.com; 10 CIBIT—Coimbra Institute for Biomedical Imaging and Translational Research, University of Coimbra, 3 000-548 Coimbra, Portugal; 11 Department of Biology and Environment, University of Trás-os Montes e Alto Douro (UTAD), Quinta de Prados, 5001-801 Vila Real, Portugal; amsilva@utad.pt; 12 Centre for Research and Technology of Agro-Environmental and Biological Sciences (CITAB-UTAD), Quinta de Prados, 5001-801 Vila Real, Portugal; 13 CEB—Centre of Biological Engineering, University of Minho, Campus de Gualtar, 4710-057 Braga, Portugal

**Keywords:** diabetes mellitus, metabolic syndrome, glucose-lowering agents, sugar-lowering oils, phytotherapy

## Abstract

Diabetes mellitus (DM) is a metabolic disorder characterized by chronic hyperglycemia together with disturbances in the metabolism of carbohydrates, proteins and fat, which in general results from an insulin availability and need imbalance. In a great number of patients, marketed anti-glycemic agents have shown poor effectiveness in maintaining a long-term glycemic control, thus being associated with severe adverse effects and leading to an emerging interest in natural compounds (e.g., essential oils and other secondary plant metabolites, namely, flavonoid-rich compounds) as a novel approach for prevention, management and/or treatment of either non-insulin-dependent diabetes mellitus (T2DM, type 2 DM) and/or Metabolic Syndrome (MS). In this review, some of these promising glucose-lowering agents will be comprehensively discussed.

## 1. Introduction

Diabetes mellitus (DM) remains as one of the major global epidemics of the 21st century. The name of this disease is derived from a Greek word meaning “going through” and a Latin word for “honey” or “sweet” [1] and is characterized by the production of a large amount of urine with a honey-like taste [2]. DM is a metabolic disorder characterized by chronic hyperglycemia together with disturbances in the metabolism of carbohydrates, proteins and fat, which in general results from an insulin availability and need imbalance [1,3,4,5]. Nowadays, the number of people suffering from DM and its complications is increasing, in part due to the current worldwide lifestyle: Sedentary lifestyle, high-fat diet, obesity and longer life span [6]. DM has become a global health problem since it is the most common clinical disorder, affecting nearly 10% of the population all over the world and constantly increasing day by day [7]. In 2015, 246 million people were estimated to have DM worldwide, among which about 80% resided in developing countries [7]. It was reported by World Health Organization (WHO) that about 1.1 million people died of diabetic complications, and it is expected that the death rate will increase up to 50% in 2030 [7]. The disease has multiple etiologies and results, briefly, from the impaired release of insulin by the pancreatic β-cells with absolute or relative insulin deficiency in plasma. Moreover, the pathophysiology of DM is also characterized by inadequate or defective insulin receptors activity, insulin-resistance characterized by an insufficient response of peripheral tissues to insulin, along with the production of inactive insulin and/or early destruction of insulin, before its action has been carried out in tissues [1,7]. As an integrated part of a metabolic syndrome, DM is a global disease with great influence on human health and mortality, particularly regarding cardiovascular disorders. Indeed, it is well-established that patients with DM also suffer from dyslipidemia and have an increased risk of coronary heart disease, as well as peripheral vascular disease and cerebrovascular disease. All together, these facts demand the control of both the blood glucose and lipid levels [7] in order to avoid the serious complications related to the disease, such as heart and blood vessel diseases [7].

DM can be manifested as type 1, also named insulin-dependent DM (T1DM or IDDM), or type 2, also called non-insulin-dependent DM (T2DM or NIDDM) [7,8]. T1DM represents about 10% of the DM cases in United States and Europe and results from the destruction of insulin-secreting pancreatic β-cells by an autoimmune-mediated process that may, under certain circumstances, have been triggered by a viral infection [3,4,8]. The pancreatic islets are infiltrated by T-lymphocytes and the autoantibodies islet cell antibodies (ICA), and insulin autoantibodies (IAA) can be detected. T1DM is subdivided in two types: T1A, also known as immune-mediated or juvenile DM is the most common form (occurs in 95% of T1DM patients) and is more prevalent among young people; and T1B or idiopathic. T1A results from the pancreatic β-cells destruction, leading to an absolute insulin deficiency, hyperglycemia and an increased breakdown of body fats and proteins. Consequently, these patients are typically lean individuals and suffer from ketoacidosis, since lipolysis is not inhibited by insulin and the free fatty acids are released from adipocytes and converted into ketones in the liver. On the other hand, idiopathic DM, is a strongly inherited DM in which there is no evidence of an autoimmune-mediated pancreatic β-cells destruction; patients suffer from recurrent ketoacidosis episodes that result from the varying degrees of insulinemia with transitory periods of absolute insulin deficiency [1]. T1DM occurs more frequently in the carriers of *HLA-DR3* and *HLA-DR4* genes, suggesting a genetic disposition for the disease [8].

T2DM is the most common presentation of DM and the fourth leading cause of death in industrialized countries, affecting over 5% of the world’s population and 1/4 of the elderly [8]. T2DM is a chronic metabolic disorder with a progressive worsening of both carbohydrate and lipid metabolism profiles. It is a heterogeneous condition characterized by a hyperglycemia status, mainly due to peripheral insulin resistance and enhanced hepatic glucose production, associated with a relative insulin deficiency. In fact, these patients can present higher, normal or low insulin levels resulting from an impaired β-cell function and insulin secretion [8,9,10]. Although T2DM is a metabolic disorder resulting from both β-cell dysfunction (with altered insulin levels) and impaired insulin action (insulin resistance), there is no evidence of human leukocyte antigen (HLA) markers or autoantibodies activity [4]. At a cellular level, the overproduction of glucose by the liver significantly leads to fasting hyperglycemia as a direct result of the increase in the excess circulating free fatty acids (FFA) being oxidized after the release from the adipocyte [8].

β-Cell dysfunction results from the (i) decreased β-cell mass, increased β-cell apoptosis or decreased regeneration, (ii) long-standing insulin resistance leading to β-cell exhaustion, (iii) glucotoxicity-inductor chronic hyperglycemia, and (iv) chronic elevation of FFA, inducing lipotoxicity and amyloid deposition in β-cells [1]. Relative insulin deficiency can also be caused by autoantibodies against insulin receptors or insulin itself, or by rare defects in the biosynthesis of insulin, insulin receptors, or intracellular transmission [11,12,13]. The following etiological factors should also be considered: (i) pancreatitis, which destroys pancreatic β-cells and leads to pancreatectomy, (ii) increased release of insulin antagonistic hormones, such as somatotropin, glucocorticoids, epinephrine, progestogens, choriomammotropin, ACTH, thyroid hormone and glucagon, and (iii) mitochondrial dysfunction (mitochondrial loss and increased production of oxidants that promote insulin resistance), since this organelle is the main source of energy to cells and thus crucial to many cellular functions, including ATP production, biosynthesis of amino-acids and lipids, cytosolic calcium transport and apoptotic stimuli control. Muscle-biopsy studies performed in T2DM patients revealed mitochondrial dysfunction and a reduced expression of peroxisome proliferator-activated receptor gamma coactivator 1 (PGC1α), which is an essential regulator of mitochondrial biogenesis and function since it interacts with co-activating transcription factors (namely, nuclear respiratory factors, peroxisome proliferator-activated receptors (PPARs), thyroid hormone and also glucocorticoid and estrogen-related α- and γ-receptors). Thus, we may consider PGC1α-regulated mitochondrial biogenesis as a possible future therapeutic target for the prevention of the mitochondrial dysfunction in diabetic patients [14].

Despite the increasing prevalence in obese adolescents, the majority of the patients belong to elderly populations and are overweight. Obesity is indeed considered a major risk factor and an important trigger for the disease [6]. Resulting from the combination of three main factors – genetic disposition, large food intake and physical inactivity—obesity leads to an imbalance between the energy supply and expenditure, increasing free fatty acids in the blood and in turn reducing glucose utilization in muscle and fatty tissues, finally contributing to insulin resistance and an increase of insulin release, further raised by the resulting down-regulation of the insulin receptors. Unfortunately, only less than one-half of the diabetic patients receive treatment and even less of these achieve glycemia levels that avoid the disease associated morbidity, thus contributing to the development of severe long-term complications at macrovascular (coronary artery disease and stroke) and microvascular (retinopathy, neuropathy, nephropathy and other microangiopathies) levels, which may have an acute or chronic character [1,8].

In fact, despite being known for decades, the cure of DM is currently an unmet clinical need. Prophylactic treatment is available and while T1DM patients require an external insulin supply since the diagnosis, initial therapy for newly diagnosed T2DM patients is conventionally an adequate diet and exercise as they lead to a marginal improvement in insulin sensitivity and a corresponding reduction in hyperglycemia [1,8]. Nevertheless, disease progression and patient non-adherence usually culminates in treatment failure within a few months and an oral antidiabetic agent is hence prescribed. Despite the great variety of therapeutic options available for hyperglycemic management in T2DM patients, including some combinations of these, they are not recognized as adequately effective in maintaining a long-term glycemic control in most patients [8,9]. Furthermore, most marketed agents are associated with some adverse events, such as hypoglycemia and/or involuntary body weight gain, that often hamper the treatment adherence. A medical need for improving pharmacological therapy of T2DM remains therefore a great need [9]. The research for new glucose-lowering drugs having minimal or no side effects and extracted from medicinal plants is a current challenge for several research groups worldwide.

There has been an increasing interest in natural compounds, especially those derived from plants, as a novel therapeutic approach to prevent, manage and/or treat T2DM and MS. In this review, novel natural sugar lowering compounds and herbal extracts currently under investigation for use in the treatment of T2DM will be discussed, considering in vitro and in vivo evidence. A special emphasis has been placed on flavonoids, naturally occurring phenolic compounds with a broad range of biological activities and whose biological effects have been increasingly studied in relation to DM. In turn, natural compounds that may be promising in the management of obesity, insulin resistance and MS, such as β-tigogenin cellobioside (tiqueside), *S. japonica* L or water-soluble chitosan, can also play an essential role in T2DM treatment. Despite the promising results, further research is required, specially focusing on toxicity, efficacy and safety studies and mechanisms of pharmacological action. The several essential oils that have demonstrated their sugar lowering capacity will be discussed in this paper.

## 2. Pathophysiology of T2DM

DM is a complex metabolic disorder with an inherent increased risk of both micro- and macrovascular disease; its main clinical characteristic is hyperglycemia [15]. In the absence of adequate interventions, the Metabolic Syndrome (MS), also called syndrome X or insulin-resistance syndrome, may lead to insulin-resistant-T2DM and related cardiovascular complications such as coronary heart disease or stroke and non-vascular pathologies such as cancer, infections, liver diseases and mental and nervous systems disorders [15]. In fact, cardiovascular diseases constitute the main cause of mortality among T2DM patients [16]. It is a dysfunction of the human metabolism essentially characterized by glucose intolerance and/or insulin resistance, hypertension, dyslipidemia and/or obesity.

**Figure 1:**
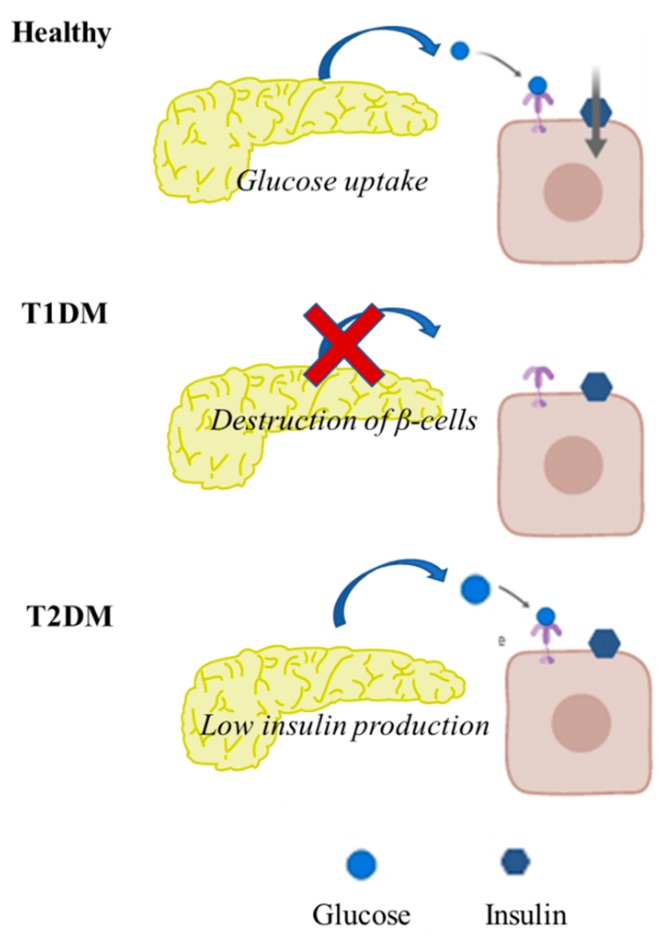
Main characteristics of pancreatic function in healthy individuals versus in diabetic individuals. In heathy individuals, the pancreas responds to hyperglycaemia with insulin secretion aiming to maintain gluco-homeostasis. Diabetes occurs when the pancreas fails to produce insulin, DMT1, insulin-dependent, or when insulin produced is insufficient or unable to produce gluco-homeostasis (DMT1).

To better understand the pathophysiology of DM, carbohydrates, lipid metabolism and their regulation, should be investigated. The main characteristics of pancreatic function in healthy individuals versus in diabetic individuals are summarized in Figure 1.

Glucose is the main substrate for energetic metabolism [17,18,19,20]. Narrow levels of plasma glucose must be maintained, by means of glucose sequestration, synthesis and consumption, being brain and erythrocytes fully glucose-dependent. Six main metabolic processes are tightly regulated in order to maintain glucose homeostasis: (i) glycolysis—glucose is converted to lactate, in erythrocytes, renal medulla and skeletal muscle (in the absence of oxygen) and in central nervous system, heart, skeletal muscle (requiring oxygen consumption), (ii) glycogenesis —glycogen is synthetized from glucose in liver and muscle, contributing to glucose storage, (iii) glycogenolysis—glucose is obtained from stored-glycogen breakdown, (iv) gluconeogenesis—glucose is produced in liver and renal cortex from amino acids, lactate and glycerol resulted from lipolysis, (v) lipolysis—glycerol and free fatty acid are obtained from triacylglycerols breakdown, and (vi) lipogenesis—fat is storage in form of triacylglycerols synthetized from free fatty acids and glycerol.

Maillard reaction products, known as advanced glycated end-products (AGEs) and as advanced lipoperoxydation end-products (ALEs), show increasing concentrations in tissue as age increases, accelerating the pathogenesis of several diabetes mellitus complications, namely, diabetic nephropathy [21]. There is an increase in lipid peroxidation and AGE/ALE compound formation in diabetic individuals, either in plasma or body tissues, most probably because they suffer a metabolic process upon both elevated glucose levels and oxidative stress status, which simulate a mechanistic connection among dyslipidemia and DM complications [22].

These facts corroborate the great need for compounds that are able to prevent the AGE/ALE formation; some of those already known (some angiotensin-converting enzyme inhibitors, angiotensin II type-1 receptor blockers, metformin, aminoguanidine, pyridoxamine, OPB-9195 and carnosine) and others that remain under research, such as LR-9 and LR-74, which have been studied in streptozotocin (STZ)-induced diabetic rat models for both premature kidney disease and dyslipidemia [21,23]. After 32 weeks of treatment, although no effect on elevated blood glucose levels was reported, there was a significant inhibition of the albuminuria, both plasma creatinine and lipid peroxidation and also hyperlipidemia; furthermore, the Nε-(carboxymethyl)lysine (CML)-AGE accumulated in the renal glomerular apparatus and tubules decreased, as well as the AGE-linked fluorescence and cross-linking of tail collagen and finally the CML and Nɛ-(carboxyethyl)-lysine (CEL) identified in skin collagen [24]. All these findings indicate that AGE/ALE inhibitors may be a good approach to retard kidney disease progression and, in parallel, prevent alterations in lipid metabolism in diabetic rat models. Finally, researchers found that these compounds may have a protective antioxidant action by inhibition of the lipid peroxidation reaction, thus providing an alternative therapeutic option to treat some diabetic macrovascular complications [22].

Several food constituents, namely, those salty, enriched with protein, having baking powder, distinct sugars and oils from a plant raw material or even grape derivatives, were analyzed for AGE inhibitor activity and it was found that (i) by using glucose as sugar, higher concentrations of CML were achieved compared to white beet sugar; (ii) refined sucrose registered much lower CML values than raw cane sugar; and (iii) the CML that is produced highly depends on the unsaturation degree of the oils, though other oil constituents (such as phenols, tocopherols, chlorophylls, carotenoid, menadione, plastochromanol-8 and oryzanols) seem to play an additional role in glycation reactions [25]. Finally, grape by-products revealed that they may function as potential antioxidants and also as therapeutic agents against bacterial colonization, obesity, thrombotic events and carcinogenic processes, mainly due to the polyphenols (PCs) and dietary fiber (DF) content [26]. Nevertheless, before these by-products become available in clinic as AGE inhibitors, there is a need for further studies regarding their toxicity, such as the presence of either residual pesticides or heavy metals [26].

When there is an increase in glycemia, glycolysis, glycogenesis and lipogenesis are increased while gluconeogenesis, glycogenolysis and lipogenolysis (mainly due to hormone action) decrease in order to restore normal glucose levels by promoting glucose reservation and degradation. When glycemia decreases, the opposite is observed [24,26]. Since lipids serve as a substrate for glucose production, DM, obesity and other metabolic conditions are straightly correlated, thus explaining why metabolic syndrome is inevitably increasing among humans [27,28,29,30].

The early onset of atherosclerosis and also the multiple mechanisms that contribute to arterial diseases in T2DM patients are closely associated with both dyslipidemia and hyperhomocysteinemia [31]. There is a correlation between the high burden of abdominal fat and the elevated levels of free fatty acids (FFA) that enrich the liver through portal circulation. This contributes to an overproduction of TG-rich lipoprotein particles, including very-low-density lipoprotein (VLDL) cholesterol (VLDL-CH) and low-density lipoprotein cholesterol (LDL-CH), a decrease in high-density lipoprotein (HDL) cholesterol (HDL-CH) and hypertriglyceridemia [7]. Besides hyperglycemia, systemic or local insulin levels elevation may contribute to abnormal lipid metabolism and a compromised endothelial function, because (i) the concentration and composition of potential atherogenic lipoproteins may be altered; (ii) changes in the apolipoprotein ratio observed in HDL can possibly interfere with its protective role in vascular disease; and (iii) insulin seems to play a direct role in blood vessel walls by increasing the levels of cellular cholesterol as it promotes an increase in cellular sterol synthesis, induction of the LDL receptors and inhibition of HDL-mediated cholesterol removal [7,31].

Nearly 80% of the T2DM patients are overweight [1]. In fact, obesity is considered a major public health problem and a relevant risk factor for numerous metabolic diseases, such as heart disease, cancer, arthritis and T2DM [16,31]. Its prevalence has been increasing worldwide with about 162 million as of 2015, and it is also of concern the increasing identification of obesity- and leanness-susceptible loci in humans [1]. Obese individuals have both an increased resistance to insulin and an impaired suppression of glucose hepatic production, leading to elevated glucose and insulin levels [1]. It was reported that a greater risk for metabolic disorders is associated with people who have upper body obesity, i.e., central obesity rather than those with lower body obesity. Peripheral resistance to insulin action and an increased production of glucose in obese people with T2DM may be due to an increased concentration of free fatty acids (FFAs), and visceral obesity is especially important because it is accompanied by increases in fasting and postprandial FFA concentrations, which contribute to: (i) stimulated insulin secretion (if acutely) or lipotoxicity (if chronic) by pancreatic β-cell; (ii) inhibited glucose uptake and glycogen storage in peripheral tissues by reducing muscle glycogen synthetase activity, leading to insulin resistance and glucose underutilization; and (iii) increased hepatic glucose production and hyperglycemia, especially in fasting plasma glucose levels, increasing the accumulation of FFAs and triglycerides that leads to reduced hepatic insulin sensitivity [1]. Thus, an increase in FFA allied with T2DM genetic predisposition in obese individuals may lead to the triad: β-cell failure, peripheral insulin resistance and abnormal hepatic glucose production.

On the other hand, low blood levels of adiponectin, an adipocytokine secreted by adipose tissue, have been shown to contribute to insulin resistance in animal models and patients with obesity and T2DM [1]. Moreover, adiponectin mRNA expression may be partially regulated by a nuclear receptor, the peroxisome proliferator activated receptor γ (PPARγ), since it regulates the expression of genes controlling FFA levels and glucose metabolism. In skeletal muscle, adiponectin increases fatty acids use as a fuel source, decreasing the tissue triglyceride content. As in MS, adiponectin levels are so low that compromise the function of this protein hormone, and adiponectin has been considered an alternatively pharmacological target to the treatment of insulin resistance. Furthermore, natural alternative agents, in the form of beverages or tea, have already demonstrated to succeed in obesity treatment, attenuating adverse effects of chemically formulated anti-obesity agents [1].

Moreover, gender differences had been observed in T2DM patients: T2DM is more frequently diagnosed in men of younger age and body mass index whereas obesity, the main risk factor, is more commonly seen in women [32]. Sex hormones have been demonstrated to play an important role in energy metabolism, body constitution, blood vessel function, and inflammatory processes.

Nutrition therapy is an important part of the treatment of T2DM, showing beneficial outcomes, together with patient education for self-management and physical activity practice, on body weight, metabolic control, and wellbeing. Thus, international guidelines have long recommended an individualized nutrition therapy for all diabetic individuals. The majority of patients may simultaneously require pharmacological therapy, which acts on at least one of several possible known targets (Table 1) [33].

## 3. Oral and Injectable Hypoglycemic Drugs Overview

### 3.1. Registered Approaches

**Figure 2:**
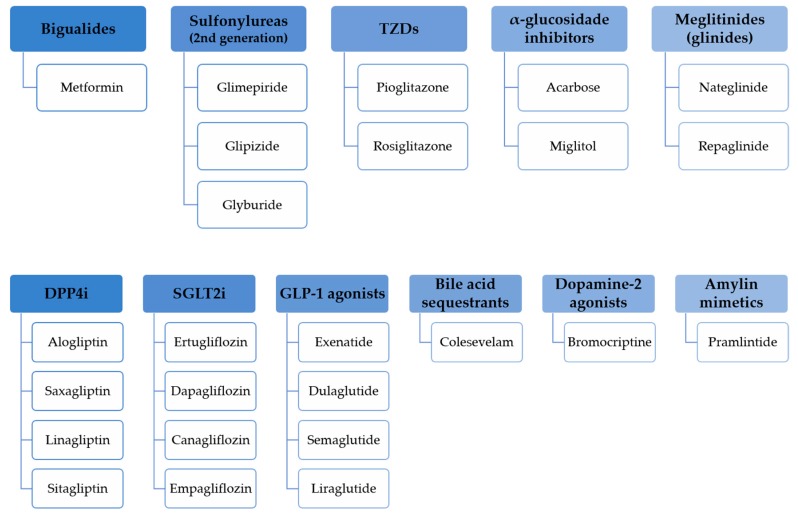
Oral and injectable non-insulin glucose-lowering drugs approved by the American Diabetes Association.

Marketed oral and injectable non-insulin antidiabetic agents (Figure 2) have been demonstrated to be effective but they possess several adverse effects that contribute to consider the diabetes treatment approach with no adverse effects a challenge.

#### 3.1.1. Sulphonylureas

Sulphonylureas have been commercially available since the 1950s and may be considered in individuals that cannot maintain a controlled diet or drug treatment other than this [23]. Sulphonylureas have a rapid intestinal absorption, thus increasing the insulin produced by the pancreas. They include glipizide, glimepiride and glyburide. When compared to first-generation, second- and third-generation sulfonylureas are able to penetrate cell membranes more promptly because of their higher lipid solubility and greater selective binding capacity [23].

As insulin secretagogues, sulphonylureas are able to reduce glycemia levels by direct stimulation of the pancreatic β-cell glucose-independent insulin secretion: they bind to one of the subunits of the K-ATP channel on the plasmatic membrane, sulfonylurea receptor type 1 (SUR1), and to the protein Epac2, enhancing the closure of the K-ATP channel, leading to cell depolarization and consequent insulin secretion [33].

Along with hypoglycemia, adverse cardiovascular effects have been found in some observational studies; findings from recent high-quality systematic reviews showed no increase in global mortality risk related to sulphonylurea use in comparison to other pharmacologic therapeutic options [23,34].

#### 3.1.2. Biguanides

Biguanides is the other class of oral anti-diabetic agents which is adequate for all types of DM. They reduce glucose absorption from the intestine, prevent liver glucose production and improve insulin’s sensitivity. Biguanides can also be used to treat mild diabetes in pregnant women [7].

Highlighting metformin, which is a guanidine synthetic analog and the orally administered drug most frequently prescribed for T2DM treatment in any age, it is known that this biguanide acts by activation of the hepatic adenosine monophosphate-activated protein kinase, thus promoting an increased glucose uptake by the hepatocytes and also a complex alteration in mitochondrial enzyme apparatus leading to gluconeogenesis inhibition [35]. In addition, it reduces free fatty acid plasma concentration, thus contributing to the gluconeogenesis reduction [36]. It was demonstrated that plasma free fatty acids largely participate in regulating glucose production in the liver. Moreover, by the reduction of glucose production rates in liver, metformin also reduces fasting plasma glucose concentrations [36]. Finally, metformin is also able to increase intestinal glucose utilization and to stimulate GLP-1 secretion [33]. Side effects of metformin are restricted to the gastrointestinal tract such as abdominal discomfort and diarrhea [31]. Lactic acidosis is rare, occurring mostly in patients with impaired renal function [31].

#### 3.1.3. Thiazolidinediones

Thiazolidinediones (TZDs) regulate gene expression, in order to enhance insulin activity in all body tissues, just by interacting with a nuclear receptor family, the peroxisome proliferator-activated receptors (PPARs) [37].

In this sense, cigliatazone, pioglitazone, troglitazone and englitazone have proved to be useful insulin sensitizing agents [38]. Activation of PPAR-γ by the thiazolidinediones contributes to maintain glucose homeostasis, improve insulin action and also prevent impaired glucose tolerance in patients [39]. Studies of the TZD action in adipose tissue have shown that they alter leptin, inflammatory molecules, and circulating proteins (such as tumor necrosis factor alpha, TNF-α) expression, thus eventually contributing to the characteristic peripheral insulin resistance [39]. Studies in animals and humans suggest that TZDs may help preserve β-cell function and thereby delay progression of T2DM [40].

Pioglitazone, a marketed TZD, is orally administered for the treatment of T2DM and contributes to increase the transcription of several regulating proteins implicated in glucose and lipid metabolisms by amplifying the post-receptor insulin action in body tissues, thus leading to an improvement in glycemic control with no associated insulin endogenous secretion enhancement. Pioglitazone co-treatment was reported to significantly decrease both HbA1c and fasting blood glucose levels in patients with a poorly controlled disease [41]. Moreover, pioglitazone has also been implicated in serum lipid profile improvement in randomized placebo-controlled clinical studies [39]. The drug is usually well tolerated by any age adult and was proven to reduce cardiovascular events [39,42].

Common side effects associated with TZDs include edema, weight gain, macular edema and heart failure. Increased bone fracture risk is another TZD-related side effect. Hepatic dysfunction (2% incidence), sometimes leading to liver failure, is a serious problem with troglitazone but not with rosiglitazone. However, rosiglitazone has been associated with an increase in myocardial infarction incidence [42]. Moreover, CYP3A4 (cytochrome P450 isoform) is induced by both troglitazone and pioglitazone, thus playing an important role in their metabolism. Safety and efficacy could be affected when these agents are co-administered with other drugs metabolized via this enzyme [42].

#### 3.1.4. α-Glucosidase Inhibitors

This class of drugs reduces intestinal glucose absorption [43] by delaying carbohydrate digestion. Highlighting acarbose, miglitol and voglibose competitively inhibit α-glucosidases, an important family of intestinal enzymes that participate in carbohydrates digestion. It is known that they decrease postprandial elevated blood glucose levels and elevated insulin, thus improving the insulin cell sensitivity and minimizing β-cells stress [43]. Additionally, these compounds have the advantage of not inducing hypoglycemia and having a satisfactory safety profile, even if associated with some gastrointestinal adverse effects that may result in difficult long-term compliance to this therapeutic option [43,44,45].

#### 3.1.5. Sodium-Glucose Co-Transporter Type 2 (SGLT2) Inhibitors

Sodium–glucose co-transporter type 2 (SGLT2) inhibitors consist of recently discovered anti-hyperglycemic agents which have a particular insulin-independent mode of action: since SGLT2s are mainly expressed in proximal renal tubules and is responsible for almost 90% of filtered glucose re-uptake, they preferably target the kidney, blocking glucose reabsorption and thus promote increased urinary glucose excretion (UGE), especially in hyperglycemic situations [9,46]. This particular mechanism of action seems to be promising for T2DM patients because patients improve glycemic control, with a low risk for hypoglycemia, and they have demonstrated benefits on weight loss, mainly due to the increased glucosuria in association with arterial blood pressure reduction, both providing an osmotic effect [9,46].

The favorable pharmacokinetic properties of SGLT2 inhibitors, namely, their excellent oral bioavailability, rather long elimination half-life that allows a single daily administration, reduced accumulation index, no active metabolites, limited renal excretion and low risk of DDIs, contribute to an increasingly interest in these compounds as oral anti-diabetic drugs [9].

Recent studies have proven that it is possible to normalize glycemic levels by inhibiting SGLT2, thereby preventing glucose reabsorption through these co-transporters and then re-entering the circulation. This insulin-independent mechanism decreases blood pressure, individual weight and plasma glucose levels without causing hypoglycemia [9,46].

Several SGLT2 inhibitors are marketed around the world, namely, dapagliflozin, canagliflozin and empagliflozin, in Europe and the United States and ipragliflozin, luseogliflozin and tofogliflozin, in Japan. Also relevant are ertugliflozin and remogliflozin, among others, which are already in the late phase of development [3,9]. They can be used either as monotherapy in diet-treated patients or combined with any other anti-glycemic agent [9].

SGLT2 inhibitors have documented a good safety profile, with low risk for hypoglycemia and high risk for both benign urinary tract and fungal genital infections [9]. Playing a vital role in carbohydrate metabolism, the kidney is responsible for the recovering the glucose presented in the glomerular filtrate under SGLT2 control, and SGLT2 inhibitors have demonstrated to be as effective as other current antihyperglycemic drugs (namely, metformin, sulphonylureas and sitagliptin). SGLT2 inhibitors may be regarded as a major approach of insulin-independent reducing hyperglycemia agents for T2DM treatment [3,9].

#### 3.1.6. Dual SGLT1/SGLT2 Agonists

A great effort has been made to discover agonists that act on both SGLT1 and SGLT2 receptors. The first developed dual combined SGLT1/SGLT2 inhibitor, sotagliflozin (LX4211), acts by blocking small intestinal SGLT1 and renal SGLT2 and SGLT1 (SGLT2, proximal convoluted tubule; SLGT1 proximal strait tubule), thus producing a diminished early phase on the absorption of glucose, reducing postprandial glycemia and insulin secretion, and increasing GLP-1 and peptide YY (PYY) circulating levels [47,48,49]. Initially, studies focused on T1DM and sotagliflozin revealed HbA1c and glucose variability reduction [47]. The program was recently extended to T2DM trials, although long-term studies will be fundamental to evaluate the risk-benefit ratio for this agent to be used as an alternative to the traditional SGLT2 inhibitors [47]. When compared to single SGLT2 inhibitors, sotagliflozin has similar potency for SGLT2 inhibition but 0.10-fold higher with respect to SGLT1 inhibition [50]. All the results indicate a great potential for SGLT1 inhibition with minimal glucosuria and an adequate therapeutic window, together with blood pressure and body weight favorable modifications [50].

#### 3.1.7. Glucagon-Like Peptide-1 amide (GLP-1) Receptor Agonists

GLP-1 receptor agonists and their analogues (incretin mimetics) constitute a new antidiabetic class of drugs that are responsible for the enhancement of glucose-dependent insulin secretion, suppression of glucagon release and pancreatic β-cells protection [46,51]. Any evidence of differences between natural GLP-1 and its analogues might probably be a consequence of the introduced modification for prolonged pharmacological action, which should be further discussed in order to improve the use and design of such analogues [51].

Injectable GLP-1 receptor agonists, such as exenatide, liraglutide, lixisenatide, albiglutide and dulaglutide, enhance insulin release induced by nutrients, inhibit glucagon secretion, delay gastric emptying and promote satiety, which lead to weight control [51]. Furthermore, an increase in β-cell mass in animals has also been reported [51].

The optimal therapeutic effect of GLP-1 agonists was found to be only achievable under provided regimens of sustained plasma levels of active peptide, which include a continuous subcutaneous infusion (CSCI) of GLP-1 and GLP-1 analogue injections at a time or two per day or even weekly with extended plasma stability [51].

Recombinant glucagon-like peptide-1 (rGLP-1) has major similarity to the native and synthetic GLP-1 sequence. In a recent study covering regimens of continuous subcutaneous infusion (CSCI) of rGLP-1, over 3 months and combined with metformin and sulphonylurea, this GLP-1 analogue was able to decrease glycated hemoglobin (HbA1c) and fasting plasma glucose, and significantly reduce body weight (sometimes not so well achieved) in a dose-dependent manner [51]. GLP-1 receptor agonists may therefore be considered a future approach in the treatment of T2DM [46,51].

#### 3.1.8. Dipeptidyl-peptidase-4 (DPP4) Inhibitors

Dipeptidyl-peptidase-4 (DPP4) is the enzyme responsible for the rapid inactivation of the incretins GLP-1 and GIP [52]. Dipeptidyl-peptidase-4 (DPP4) inhibitors, namely, linagliptin, vildagliptin, sitagliptin, saxagliptin, and alogliptin, are once or twice-daily oral drugs which enhance endogenous effects of incretin by means of DPP4 inhibition, thus retarding incretin degradation and prolonging their circulating half-lives [46].

#### 3.1.9. Bile Acid Sequestrants

Bile acid sequestrants (BAS) are considered a good approach to T2DM treatment since dyslipidemia is known to exacerbate T2DM-related cardiovascular complications and these compounds have both glucose and lipid lowering action in clinical trials [53]. BAS produce a nonabsorbable complex that avoids bile acid reabsorption, thus increasing its intestinal excretion [54]. This, in consequence, promotes more bile acids to be synthetized and transforms low-density lipoprotein (LDL) cholesterol into bile acids, promoting a reduction in plasma LDL levels [54]. A systematic review regarding meta-analyses of randomized controlled trials showed that diabetic patients treated with BAS reported a glycemic control improvement (HbA1c reduced 0.55% in end of treatment trials and 0.40% in mean change trials) and reduction on LDL cholesterol levels (not so significant as with statin treatment), with no apparent influence on weight [54]. Some gastrointestinal adverse events (constipation was the most common) and triglyceridemia elevation were observed but not significant to display clinical evidence [54]. Colesevelam (second-generation) was the only BAS approved by the US Food and Drug Administration (FDA) for hyperglycemia treatment in T2DM patients, in 2008 [53,54].

#### 3.1.10. Dopamine Type 2 Receptor Agonists

Bromocriptine (BC), a dopamine type 2 receptor (D2R) agonist, was the first approved and patented dopaminergic drug for T2DM treatment, by the FDA in 2009 (quick-release formulation- bromocriptine-QR) [55,56,57]. Several tests have been performed since then. For instance, in an alloxan-induced diabetic rat model, BC anti-hyperglycaemic effect was tested, either with or without concomitant glipizide: when administered alone, bromocriptine was found to lower glycemia values and this result was several times potentiated with glipizide co-administration, thus indicating that bromocriptine must be considered for adjuvant anti-diabetic therapy [55]. Another study was performed administrating BC-QR in D2R knockout mutant mice, with glucose intolerance and impaired insulin secretion, revealed fasting glucose, lipolyisis and lipogenesis reduction and less severe adverse effects and cardiovascular disease manifestations [55,57]. In obese men, weight loss, body fat loss and glucose tolerance improvement were reported [57]. Thus, bromocriptine decreases insulin resistance and glucose synthesis in the liver by potentiating dopaminergic neurotransmission which in turn resets the hypothalamus and enhances insulin sensitivity in body tissues [55]. Further clinical trials must be performed to confirm the above-mentioned findings, and although a protective effect of BC on pancreatic beta-cells endogenous insulin secretory capacity has been reported, the FDA does not recommend its use in T1DM [57]. Finally, this evidence indicates that insulin secretion is not only glucose-regulated but is also controlled by parasympathetic or sympathetic input, especially from the dopaminergic system [57].

#### 3.1.11. Amylin Mimetics

Amylin is a ~4 kDa peptide hormone co-secreted by pancreatic beta-cells with insulin and is responsible for maintaining glucose metabolism homeostasis [58]. It acts by activation of amylin receptors, some of them shared with calcitonin gene-related peptide (CGRP). This peptide modulates ingestion (induces satiety) and reduce both insulin secretion and sensitivity although with a short half-life of 13 min [58,59,60]. Amylin mimetics have been developed regarding weight loss in overweight diabetic patients, usually participating in a combined therapy approach where leptin seemed to be a good option: amylin may enhance leptin sensitivity and prevent leptin resistance [59]. At the moment, pramlintide is the only one available on the market for clinical use in both T1DM and T2DM. Next-generation amylin mimetics with improved pharmacokinetics have been developed as anti-obesity strategies: (i) davalintide, a much more potent and effective agent, with greater affinity to human CGRP receptor and a prolonged action; (ii) PEGylated or glycosylated amylin, whose chain was modified by either polyethylene glycol (PEG) coupling or glycosylation, thus having a greater half-life (~10 h 15); and finally (iii) dual amylin and calcitonin receptor agonists (DACRA), KBP 042, KBP 088 and KBP 089, which bind to both amylin and calcitonin receptors and produce a greater eating inhibition [59].

### 3.2. Novel Approaches

Synthetic compounds seem to be promising future treatment approaches in T2DM and MS treatment.

#### 3.2.1. Mangiferin-Berberine Salt

Mangiferin is a xanthone glycoside extracted from *Mangifera indica* and *Anemarrhena asphodeloides*. It has hypolipidemic, hypoglycemic, insulin-sensitizing, anti-obesity, antioxidant and anti-inflammatory properties in animal models and also in clinical studies, probably due to the activation of AMP-activated protein kinase (AMPK) [41]. As a result of this activation, the energy balance can be controlled and hence, lipid and glucose metabolisms [41]. Berberine is an isoquinoline alkaloid isolated from *Coptis chinensis* that seems to be a promising agent in modulation of both carbohydrate and fat metabolisms, by means of LDL receptor upregulation, modulation of the intestinal microbiota and the activation of AMPK [41].

Mangiferin-berberine salt is a synthetic compound obtained from mangiferin (M) and berberine (B) chemical bonding at an equal molecular ratio; it presents an acidic M group conjugated with an alkaline B group by ionic bond forming a stable single molecule [41]. As M and B roles in the improvement of metabolic disorders are undeniable, it was thought that MB salt could also have beneficial modulatory activities in carbohydrate and fat metabolisms [40]. In fact, recent pilot studies demonstrated that the mangiferin-berberine salt (1) stimulates the AMPK signaling pathway in L6 skeletal muscle cells, (2) lowers both glucose and lipids levels in blood and (3) improves insulin sensitivity and liver function in KK-Ay diabetic mice models [41]. Furthermore, efficacy studies performed on the HepG2 cell line corroborated that MB salt truly has a potent stimulating activity in AMPK and in modulating carbohydrate and fat metabolisms; these properties have shown to be better than the ones found for M or B alone [41].

MB salt is therefore a promising antidiabetic agent as it is able to (1) act as a potent AMPK activator, suppressing gluconeogenesis, (2) prevent hepatic steatosis and reduce the triglyceride (TG) content in hepatocytes, (3) suppress the upregulation of lipogenic and diabetic genes, attenuating lipogenesis, (4) stimulate basal and insulin-stimulated glucose consumption, (5) stimulate glucose metabolism having no insulin, (6) inhibit glucose production and (7) downregulate PEPCK/G6Pase, without cellular toxicity and in a dose-dependent manner [41].

#### 3.2.2. β-tigogenin Cellobioside (Tiqueside)

Saponins, either natural or synthetic, can function to reduce cholesterol absorption and plasmatic levels, having an increasing interest in hypercholesterolemia management [61]. Aiming at determining the properties of saponins in lipid metabolism, a study was carried out using the synthetic soponin β-tigogenin cellobioside, also named tiqueside or CP-88818, on male golden Syrian hamsters [61]. It was found that tiqueside could reduce cholesterol absorption, depending on the administered dose, either in the presence or absence of exogenous cholesterol [36]. In addition, the administration of tiqueside to chow-fed hamsters contributed to decrease intestinal cholesterol absorption without any alterations in bile absorption or 7α-hydroxylase cholesterol activity, thus suggesting no interference in enterohepatic bile acid recirculation [61]. Furthermore, cholesterol levels in liver were reduced in a dose-dependent manner and were correlated with an inhibition of cholesterol absorption; an induced compensatory increase in the activities of both intestinal HMG-CoA reductase and hepatic LDL receptor levels were also registered [61]. Among other animal species, tiqueside also produced plasma cholesterol lowering, either with diets containing cholesterol (hamster, rat, mouse and dog) or diets without it (hamster, rat, rabbit, mouse, cynomolgus monkey, rhesus monkey and SEA quail), which suggests a broad action [61]. Except for dogs, plasma cholesterol reduction was mainly due to blood non-HDL cholesterol lowering levels with a small or even no alteration in circulating HDL cholesterol levels.

Tiqueside inhibits cholesterol absorption, producing large effects on cholesterol metabolism, leading firstly to a reduction of circulating non-HDL cholesterol, with no alteration in bile acid metabolism [61]. Synthetic saponins, such as tiqueside, may thus be regarded as a potential treatment of hypercholesterolemia and in this way, also contributing to metabolic syndrome and T2DM management [61].

#### 3.2.3. Incretin-Based Therapies

Insulin secretion is admittedly much greater after glucose oral intake than after endovenous administration, which may indicate that insulin is secreted by means of stimulating factors related to sugar ingestion, incretins, that act as intestinal tract messengers [15]. Incretins are a group of metabolic hormones that share a postprandial hypoglycemic effect and an enhancement of insulin secretion by pancreatic β-cells by means of a glucose-dependent mechanism, and that also inhibit glucagon secretion by α-pancreatic cells [62]. The two main molecules belonging to this class are the intestinal peptides glucagon-like peptide-1 amine (GLP-1) and glucose-dependent insulinotropic polypeptide (GIP), although T2DM patients seem to have no major GIP secretory defect [15,63]. Both of them are inactivated by the enzyme dipeptidyl peptidase-4 (DDP4) very rapidly [63]. Incretin-based therapies have huge potential in T2DM treatment [46,63]. Among the available therapies, three of them dominate, namely, glucagon-like peptide-1 amide receptor agonists, dipeptidyl-peptidase-4 inhibitors and Takeda-G-protein-receptor-5 (TGR5) agonists [46].

#### 3.2.4. TGR 5 Agonists

TGR5 bile acid receptors, also named G protein-coupled bile acid receptor 1 (GPBAR 1), G-protein-coupled receptor 19 (GPCR19) or membrane-type receptor for bile acids (M-BAR), are membrane receptors specific to bile acids, present in L cells, that stimulate GLP-1 secretion [46].

Preliminary studies are being performed in order to evaluate if TGR5 agonists, which are barely absorbed, may act further along the intestinal tract, mainly in the ileum, to enhance GLP1 secretion by activating these TGR5 receptors in L cells, as it has been demonstrated in other performed studies with colesevelam, a bile acid sequestrant indicated as a glucose-lowering drug in some countries [46].

TGR5 agonists may be then considered promising antidiabetic agents and a future approach for T2DM drug therapy [46].

#### 3.2.5. β-Cell Acting Compounds

Since there is a great need for lasting efficacy interventions able to prevent, attenuate and/or reverse pancreatic β-cell dysfunction and β-cell mass reduction in T2DM patients, compounds found to act in pancreatic β-cells may be considered an excellent approach for drug therapy [46].

Four classes, namely, small molecule insulin releasers, glucokinase activators, fatty acid receptor agonists and imeglimin, are currently under investigation and will be discussed in the next sections [46].

#### 3.2.6. Small-Molecule Insulin Releasers

Small molecule insulin releasers, namely, succinate esters, imidazolines, selective phosphor-diesterase inhibitors, antagonists of α2 adrenergic receptors, and agents that close potassium channels or open membrane calcium channels, have shown to improve β-cell function in vitro, by a mechanism similar to that of current antidiabetic agents, such as sulfonylureas and meglitinides. However, it is important to note that in vivo experiments have demonstrated that these promising compounds are non-specific in targeting pancreatic β-cells [46].

Further investigation is thus required for a better understanding of their biological targets and possible biological effects.

#### 3.2.7. Glucokinase Activators

The action of glucokinase, a glucose phosphorylating enzyme, can be enhanced by glucokinase activators [46]. Glucose phosphorylation prevents glucose leak from the cell and prepares it for further metabolism. Glucokinase present in pancreatic islet β cells functions as a glucose sensor. Additionally, it plays also an important role in both hepatic glycogenesis, where small molecules of glucose are joined together and converted into a glycogen molecule, and liver gluconeogenesis regulation [64]. Therefore, glucokinase activators lead to an increase in insulin secretion and also in hepatic glucose metabolism, thus, decreasing blood glucose levels [46]. When stimulating insulin secretion at low glucose levels, they seem to promote hypoglycemia [46].

Since glucokinase has a different regulation mechanism in liver cells compared to that in the pancreas, future investigations should focus on liver-selective glucokinase activators and compounds that also share an ability to enhance futile cycling of hepatic triglycerides, during a prolonged action of glucokinase in the liver [46].

Moreover, recent investigations using mice genetically modified for glucokinase and also those in mutated humans have highlighted how glucokinase is relevant in maintaining carbohydrate homeostasis, thus considering that pharmacotherapies that enhance glucokinase biological activities may be a promising approach for T2DM treatment [65].

#### 3.2.8. Fatty Acid Receptor Agonists

Free fatty acids can function as a ligand for several G-protein coupled receptors (GPR) [46].

Once activated, the receptors are expressed in pancreatic β-cells, e.g., GPR40 (also referred as FFAR1) and GPR119, contribute to enhance insulin secretion; although, as they are also expressed in α-cells, they contribute to an undesirable glucagon enhanced secretion [46].

These receptors are also expressed by other cells, such as other enteroendocrine pancreatic cells, K cells, L cells and I cells [46].

With respect to the mechanisms of action, while GPR40 agonists increase calcium concentration in the cytosol, stimulating insulin secretion, GPR119 agonists activate adenylate cyclase cascade leading to augmented levels of adenosine monophosphate and the potentiation of nutrient-induced insulin secretion [46].

Furthermore, the receptor GPR120, which is mainly expressed by adipocytes, promotes adipogenesis [46]. Synthetic agonists for the referred receptors lead to an increased secretion of several hormones, such as GIP, GLP-1, peptide YY (PYY) and cholecystokinin (CCK), thus potentiating the incretin and satiety effects of these hormones, and also increase insulin sensitivity [46].

#### 3.2.9. Imeglimin

Imeglimin, a triazine derivative, stimulates glucose-induced insulin secretion and improves glycemic control in T2DM patients. It is believed that this compound may change cellular energetics, partially due to a closure of mitochondrial permeability transition pores, and also improve insulin sensitivity at the periphery and reduce hepatic gluconeogenesis [46].

#### 3.2.10. Adipokine-Based Treatments

There are several adipocyte hormones that play an important role in energy metabolism. Leptin stimulates insulin and inhibits glucagon actions, besides promoting weight loss by means of central satiety and thermogenic effects [66,67]. However, the effects of this hormone and its analogues could only be achieved in T2DM patients who suffer from severe leptin deficiency [46].

Adiponectin, usually at low concentrations among T2DM patients (especially in overweight ones), is responsible for insulin sensitivity improvement, endothelial function enhancement and an anti-inflammatory effect. Therefore, in recent studies, the orally active adiponectin receptor agonists, ADIPOR 1 and ADIPOR 2, have been demonstrated to have glycemic control improvement and the extension of the lifespan of insulin resistant diabetic animals, being regarded as new promising agents for clinical trials [46].

Retinol-binding protein 4 (RBP4) is a plasma retinoid transporter detected in the early phase of insulin resistance conditions, such as in T2DM and MS; its inhibition allowed an increase in insulin sensitivity in animal trials [46].

Fibroblast growth factor 21 (FGF21) may be considered as a distinct member of the FGF family due to its properties in the endocrine system. Although with a predominant hepatic expression, it is also found in the pancreas and adipose tissue. Pharmacological a peptide secreted by adipocytes, hepatocytes and myocytes, contributes to fatty acid β-oxidation and gluconeogenesis in the liver during starvation. Since FGF21 is elevated in the plasma of T2DM patients and obese individuals, mainly due to FGF21 resistance, the administration of FGF21 analogues seems to be a favorable approach as they probably will lead to an improvement of the lipid profile, reduction of insulin resistance and glucose-lowering, in part due to the increased adiponectin production [46].

#### 3.2.11. Selective Peroxisome Proliferator-Activated Receptor (PPAR) Modulators

PPAR is a family of nuclear receptor proteins that function in transcription in cells, playing an essential role in regulating cellular differentiation, development and carbohydrate, lipid and protein metabolism [46] There are three types currently well identified: (i) PPARα, responsible for the improvement of the lipid profile, inflammation process attenuation and management of microvascular complications, although it is known that it may raise creatinine levels and the risk for myopathological abnormalities, (ii) PPARβ/δ, which contributes to weight gain by increasing thermogenic energy expenditure, and (iii) PPARγ, which leads to insulin sensitivity improvement, glycemic control and participates as a vascular health marker, also reducing inflammation, but it also contributes to increase fluid retention and heart failure risk, bone mineral density reduction and enhanced adipogenesis [46].

Although they have shown promising therapeutic effects, PPAR selective agonists—dual PPARα/γ agonists (or glitazars) and triple PPARα/γ/δ agonists (or panPPARs)—were not applied in clinical use due to the reported side effects, which need further investigation to be minimized [46]. For this reason, selective PPAR modulators (SPPARMs) have been designed in an attempt to overcome those undesirable effects. An example of success in recent clinical trials is INT131, a non-thiazolidinedione that has demonstrated glucose-lowering efficacy with less edema and less weight gain when compared with pioglitazone [46].

#### 3.2.12. β-hydroxysteroid Dehydrogenase 1 (11βHSD1) Inhibitors

11β-hydroxysteroid dehydrogenase 1 (11βHSD1), also known as cortisone reductase, is an NADPH-dependent enzyme responsible for reducing cortisone to the active hormone cortisol, which activates glucocorticoid receptors, being expressed in key metabolic tissues, namely, the liver, adipose tissue and central nervous system [46]. 11βHSD1 inhibitors thus lead to a diminished active cortisol production [46]. For instance, a recent 12-week randomized phase 2 double blind placebo-controlled study using INCB13739 in metformin-treated T2DM patients showed an insulin sensitivity improvement, HbA1c reduction, lipid profile improvement and body weight reduction [46].

Apart from the theoretically promising therapeutic effects in lowering cortisol levels, 11βHSD1 inhibitors were of low efficacy since a circulating cortisol reduction may occur, thus promoting a compensatory adrenocorticotropic hormone increased secretion [46].

#### 3.2.13. Antiobesity Drugs

Since obesity is the major risk factor for T2DM development, there is an evidence that drugs currently prescribed for weight loss can also contribute to glycemia control [46].

Orlistat, an intestinal lipase inhibitor, and other new satiety-inducing drugs, such as liraglutide (high-dose GLP1 receptor agonist), lorcaserin (5-HT2c serotonin receptor agonist) and combinations of phentermine-topiramate or bupropion-naltrexone have demonstrated a great potential in T2DM treatment [68]. There are further weight-lowering drugs, mainly intestinal satiety hormones, under investigation and early development [46].

#### 3.2.14. 55.P0110

For a long time, traditional herbal remedies served as a source of chemical leading structures for the design of superior synthetic analogues, compounds that can be traced back to a scaffold of natural origin and thus serving as an initial lead for development. This has also been true for diabetes mellitus treatment [69].

For instance, lupins used in Mediterranean traditional ethnic medicine, have been employed to treat diabetes mellitus regarding their antidiabetic activity, due to their abundant quinozolidine alkaloid content [69]. These compounds have proven to produce glucose-lowering effects and have a direct insulinotropic action on pancreatic islets when feeding diabetic animals with extracts from *Lupinus termis* or *Medicago sativa;* lupin alkaloids-rich plants demonstrated an improvement in hyperglycemia, hypercholesterinemia and DNA damage [69].

55P0110 is one of these synthetic compounds that has been tested for potential antidiabetic activity [70]. Despite no evidence of a direct molecular target, it is believed that this compound enhances glucose-stimulated insulin release [69]. Since a low potential has been reported for hypoglycemia and 55P0110 has proven to be as effective as current established oral antidiabetic drugs, next studies are expected to focus on its mechanism of action, i.e., whether the action of this new compound involves effects on membrane or nuclear receptors of pancreatic β-cells, such as GLPR1, GIPR and GPR40, and/or on their endogenous agonists, thus potentiating glucose-induced insulin secretion [69]. Oral bioactive derivatives of (-)-multiflorine previously studied allowed the discovery of a novel class of fully synthetic substituted quinazolidines, which have demonstrated blood glucose lowering properties [69]. Combined together, all the distinct glucose lowering potential, low risk for fasting hypoglycemia and attractive pharmacological profile of 55P0110 and analogues, further studies are recommended in order to discover structures for potential antidiabetic drugs [69].

#### 3.2.15. Antiosteoporotic Trace Minerals: Silicon (Si) and Strontium (Sr)

Previous studies on soluble silicon (Si) to understand its action on bone metabolism and inhibitory effect on PPARγ have been reported. PPARγ participates either in glucose or bone metabolism, increases adipogenesis at the expense of osteogenesis, thus contributing to bone loss [71,72].

A study on obese diabetic KK-Ay mice was carried out to evaluate the anti-diabetic potential of bone-seeking Si and stable strontium (Sr), having coral sand (CS) as the natural material containing the referred elements [72]. It was found that after 56 days of been fed diets containing CS, there was a great decrease in plasma glucose, insulin, leptin, and adiponectin levels and, on the other hand, a significant increase in pancreatic PPARγ and adiponectin mRNA expression levels, thus leading to an improvement of the β-cells’ glucose sensitivity and a decrease in insulin expression [72]. Furthermore, it has been demonstrated that there was an improvement in renal PPARγ, PPARα and adiponectin expression, histologic indices of diabetic glomerular lesion and plasma renal function indices [72].

Taking these results into account, it can be concluded that anti-osteoporotic trace minerals such as Si and Sr, due to their demonstrated lowering effect in glycemia, contribution to improve insulin, leptin, and adiponectin tolerance and lowering glomerulopathy risk through modulation of related pancreatic and renal gene expression, can be regarded as a promising approach to T2DM treatment [72].

#### 3.2.16. Hybrid Molecules and Hybrid Natural Products

There has been an increasing interest in producing hybrid molecules in order to combine all the beneficial properties and/or roles of several compounds into a single compound [71]. Recent studies tried to assess the behavior of hybrid peptides such as GLP1 linked with glucagon, GIP or any other intestinal peptide, in an effort to lower blood glucose levels in T2DM patients [73]. Thus, it is possible to combine the effects of a great variety of peptides, such as incretins, glucagon receptor agonists or antagonists, oxyntomodulin, PYY, obestatin and ghrelin antagonists, for blood glucose and lipid levels, satiety, energy expenditure and adiposity, among others [71].

In spite of some physicochemical constraints and the need to address some potential immunological issues, since these hybrids can be produced with desired sequences to give rise to chimeric molecules that interact with specific epitopes and allow novel therapeutic targets in only a single molecule, multipurpose designer molecules offer a promising approach in the therapy of several conditions, namely, T2DM [71].

For the same reason, hybrid natural products, such as O-alkylated xanthone, carbazoles and coumarins, have been recently produced. Their anti-diabetic activity has been investigated in vitro regarding their action as inhibitors of the enzymes glucose-6- phosphatase, glycogen phosphorylase and α-glucosidase, which are major checkpoints of the sugar metabolism regulation and seem to be raised in DM [71]. Inhibiting glucose-6-phosphatase at the penultimate stage of gluconeogenesis decreased the hepatic glucose output, thus lowering glucose plasma concentration. On the other hand, inhibiting glycogen phosphorylase that contributes to the glucose release from glycogen diminished the energy substrate providing for a number of pathways, namely, glycolysis. Finally, inhibiting α-glucosidase, a membrane bound enzyme found in small intestine epithelium, glucose cleavage from disaccharide is reduced, thus leading to deficient production of monosaccharides [71]. Compounds found to be significant inhibitors were further selected for in vivo testing to evaluate their glucose-lowering effect in both sucrose-loaded normal and STZ-induced diabetic rats [71].

Although they have obtained some interesting results, there is a need for further investigation, since these hybrid compounds seem to be a future natural approach in T2DM treatment and management [71].

#### 3.2.17. Bis(α-furancarboxylato)oxovanadium (IV) (BFOV)

Vanadium compounds are nonspecific phosphotyrosine phosphatase inhibitors that have proven insulin-mimetic and/or insulin enhancing activity either in vitro or in vivo, countering hyperglycemia observed in T1DM human subjects and also maintaining glucose homeostasis in T2DM subjects [74,75].

The inorganic vanadium salts, vanadyl sulfate and sodium metavanadate, were excluded from clinical trials in later phases because of their evidenced low bioavailability and digestive tract irritation [74]. To be considered an orally delivered hypoglycemic agent, vanadium needs to cross biological membranes in order to be absorbed and undergo intracellular uptake. By means of a metallocomplex (complex state of vanadium), most metal ions can pass through cell membranes by passive diffusion, since this transporter has low molecular weight, neutral charge, and proper resistance against hydrolysis and lipophilicity-hydrophilicity balance [74]. Taking these findings and compared to inorganic vanadium salts, vanadium complexes with organic ligands seem to have large biological activity with low toxicity [74].

Bis(α-furancarboxylato)oxovanadium(IV) (BFOV) is an organic vanadium complex found to lower glycemia, intolerance to glucose along with hyperinsulinemia and also to promote insulin sensitivity, activate glucokinase, increase the glycogen content in the liver and suppress phosphoenolpyruvate carboxykinase hepatic and renal gene expression in recent studies using fat-fed/ streptozotocin-diabetic rats [74]. BFOV may thus promote an increase in hepatic glucose disposal and ensure carbohydrate homeostasis [74].

Furthermore, BFOV holds an almost perfect combination of pharmacokinetic properties, such as good water solubility, moderate hydrolytic stability and great lipophilicity, which allow this compound to be regarded as a pharmaceutical agent [74].

Since BFOV has demonstrated anti-diabetic and insulin-sensitizing activities in diabetic rats, it should be regarded as a new orally active anti-diabetic organic vanadium complex that may be considered as a promising new therapeutic agent for T2DM treatment [74].

#### 3.2.18. LR Compounds: LR-9 and LR-74

As mentioned in the Introduction of the present paper., the production of Maillard reaction products (AGEs/ALEs) is a current clinical concern as they are implicated in the pathogenesis of several diabetes mellitus complications [22].

Therefore, a great effort in proposing new AGE/ALE formation inhibitors, either of natural or synthetic origin, has been made. Besides all the ones mentioned in Section 1, LR-9 and LR-74 were recently studied using a streptozotocin (STZ)-induced diabetic rat model [22]. After 32 weeks of treatment, it was found that there was a significant inhibition of the albuminuria, creatininemia, hyperlipidemia and lipid peroxidation in the plasma, but no considerable effect on hyperglycemia. It was also noticed a reduction in (i) renal glomerular and tubular accumulation of CML-AGE, (ii) AGE-related fluorescence and tail collagen cross-linking, and (iii) skin collagen CML and CEL levels [22]. All these facts suggest that LR-9 and LR-74 may be able to prevent both nephropathy progression and dyslipidemia, having an additional antioxidant effect on lipid peroxidation, providing an alternative therapeutic option to treat diabetic macrovascular complications [22].

#### 3.2.19. Insulinomimetic Zinc (II) Complexes with Natural Products

Zn(II) ion, an essential trace element, is found in hundreds of metalloproteins and active sites of several metalloenzymes and was first reported as an insulin-mimetic agent in 1980, by Coulston and Dandona [76]. Further investigation allowed researchers to regard Zn(II) complexes with Zn(O_4_), Zn(N_2_O_2_), Zn(N_2_S_2_), Zn(O2S2) and Zn(S4) as potent insulin-mimetic agents and thus establish blood glucose level reduction in T2DM patients [76]. Finally, in further performed studies, using KK-Ay mice, it was reported that Zn(II) complexes with natural products, such as Zn(On) combined with betaine (bet), L-lactic acid (lac) and D-(–)-quinic acid (qui), were able to produce glucose-lowering effects [73]. Finally, these findings were well observed for Zn(II) complexes with lactic and quinic acids, Zn(lac)2 and Zn(qui)2, respectively [76].

These findings thus suggest that Zn(II) complexes with natural products may be regarded as a future T2DM treatment approach, although there remains a great need for further investigation.

#### 3.2.20. 4-(2,2-dimethyl-L-oxopropyl)benzoic Acid

Administrating the compound 2-(1,1-dimethylethyl)-2-(4-methylphenyl)-1,3-dioxolane showed reduced glucose plasma levels and fatty acid β-oxidation (FAO), by actively sequestering the intramitochondrial CoA required for fatty acids transport into the mitochondria [8]. However, this mechanism of CoA sequestrating seems to be harmful to tissues other than the liver that also depend on acyl-CoA intermediates processing, thus producing cellular toxicity [8]. Therefore, a prodrug was developed in order to increase drug bioavailability at a sufficient rate to inhibit FAO, but easily conjugated and eliminated by hepatocytes, through the bile, in order to avoid harmful concentrations in the bloodstream [46]. This was achieved by protecting the function of the 4-(2,2-dimethyl-L-oxopropyl)benzoic acid in a way that only after the cleavage by hepatic nonspecific esterases or P450 enzymes the prodrug becomes the active compound, thus performing its activity [8].

Several polyol esters and ethers of ketone were employed in order to better modulate the targeting and release of the active pharmacophore [8]. In future research regarding human subjects, it is important to note that ester prodrugs may be species-independent and thus their biological activity is similar either in animals or in human subjects; in turn, ether prodrugs and other more extensively modified compounds may be demonstrate some differences among species, a fact that may affect prodrug activation rate and its reported toxicity effects [8].

Hence, 4-(2,2-dimethyl-L-oxopropyl) benzoic acid may be regarded as a novel pharmacological agent for T2DM and metabolic syndrome treatment, since it can undergo hepatic activation without releasing the active pharmacophore into the bloodstream and thus reducing blood glucose levels by inhibiting FAO-dependent gluconeogenesis, without any evidence of toxicity [8].

#### 3.2.21. Boronated Nucleosides and Nucleotides

Base-boronated nucleoside and phosphate-boronated nucleotides were found to be good hypolipidemic agents in recent animal experiments, since they could lower serum cholesterol and triglyceride levels, reduce VLDL and LDL cholesterol levels and elevate HDL cholesterol levels, reduce cholesterol, triglyceride and phospholipid levels in tissues, suppress appetite and reduce phosphatidylate phosphohydrolase activity, presenting with low toxicity values [77]. They may thus be regarded as promising agents in T2DM and metabolic syndrome treatment and management.

## 4. Natural Sugar Lowering Compounds

### 4.1. Diplacone and Mimulone

Diplacone and mimulone are two geranylated flavanones that had anti-inflammatory, antiradical, cytoprotective and antibacterial activities in recent in vitro studies and also in an alloxan-induced diabetic mice model. Diplacone exhibited a cytoprotective effect and a scavenger action on superoxide anion and hydrogen peroxide [78]. This has been suggested to result from the structural characteristics of the flavonoid skeleton of diplacone, such as the substitution of 5,7-dihydroxy, 4-oxo and 3′,4′-dihydroxy (catechol moiety) on rings A, C and B, respectively [78]. The best activity of diplacone may be due to the presence of the catechol moiety while the mimulone scavenging ROS activity may be due to the single hydroxyl group (para-hydroxyl) present on ring B [78].

Furthermore, studies in colitis-induced Wistar rat model diplacone evidenced the best treatment profile, with the lowest disease activity index at the last moment of the experimental procedure. Both compounds also ameliorated and delayed alterations in stool consistency and rectal bleeding because of their potential antioxidant activity, scavenging ROS and interacting with antioxidant enzymes, such as superoxide dismutase-2, catalase, cyclooxygenase-2 (COX-2) and matrix metalloproteinase-2 (MMP-2), responsible for antioxidant defensive mechanisms [43]. Unfortunately, the tested compounds could not fully prevent the histological damage and marked shortening of the colon [78]. Other flavonoids sharing an anti-inflammatory effect, namely, hesperidin, chrysin, luteolin, icariin and EGCG, should also be tested [78].

Flavonoids share a great potential as novel anti-inflammatory drugs and they can be thought of as new therapeutic approaches against T2DM and/or MS, essentially due to their potential anti-inflammatory activity [78].

### 4.2. Ethanolic Extract of Semecarpus Anacardium (Linn.) Bark

*Semecarpus anacardium* (Linn.), usually known as Beula (Bangla), Bhallataka (Sanskrit) or Marking nut tree (English), has been often used in traditional medicine for the treatment of gout, rheumatic pain and cancer, due to its immunomodulatory, anti-inflammatory, anti-arthritic and anticancer activities mediated by the rich content of phenols, such as carbolic acid derivatives, bhilawanols, sterols, glycosides, anacardic acid, anacardoside and flavonoids [7,79]. *S. anacardium* nuts have demonstrated activity in lowering blood glucose and cholesterol levels and also as antitumor, antioxidant, cytotoxic, fungistatic, anti-lipid peroxidative and liver protective agents [7].

A recent study aimed at determining the effects of ethanolic *S. anacardium* stem bark extract (i) on circulating glucose, hepatic enzymes aspartate aminotransferase (AST/GOT) and alanine aminotransferase (ALT/GPT), total cholesterol (TC), triglycerides (TG), LDL- and HDL-cholesterol levels, (ii) on hepatic glycogen content, and (iii) as an antioxidant agent in an alloxan-induced diabetic rat model [7].

The obtained results showed (i) 100% survival rate in rats administrated with the highest concentration of the ethanolic extract, (ii) dose-dependent blood glucose lowering level for all tested groups, (iii) no meaningful alteration in the ratio of organ weight to body weight, (iv) significant anti-diabetic effect accompanied by a dose-dependent TC, TG, LDL lowering effect, and (v) assured protection of the liver, explained in part by the reduction of GOT and GPT levels and the increase in hepatic glycogen [7]. The reported biological activities may be due to rich content of steroids, triterpenoids, flavonoids, glycosides, saponins and tannins [7].

The ethanolic extract of *S. anacardium* stem bark presents a higher phenolic and flavonoid contents, exhibiting antidiabetic and antioxidant activities, both comparable to metformin (commercial antidiabetic drug) and ascorbic acid (antioxidant) [7]. For this reason, the extract of *S. anacardium* can be used as a natural antidiabetic and antioxidant agent, supporting its traditional use in the treatment of diabetes mellitus and as a natural source of antioxidants [7].

### 4.3. Catalpol

Catalpol is an iridoid glycoside that exists in *Radix rehmanniae* roots [14]. It showed a remarkable hypoglycemic effect in a streptozotocin-induced diabetic model, although the underlying mechanism of action is not yet fully understood [14]. It was also reported that catalpol is able to: (i) increase the expression of glucose transporter subtype 4 (GLUT 4) in skeletal muscle, (ii) ameliorate diabetic encephalopathy (iii) protect neurons against H_2_O_2_-induced apoptosis, (iv) participate in some anti-inflammatory mechanisms, (v) decrease the alterations observed in mitochondrial function, since it enhances complex I biologic action and mitochondrial membrane potential, and finally (vi) decrease ROS generation [80,81].

Since mitochondrial dysfunction participates in the physiopathology of T2DM and the improvement of mitochondrial function may contribute to a novel T2DM therapeutic approach, a study evaluated the effect of oral catalpol in muscle mitochondria of high-fat diet/streptozotocin (HFD/STZ)-induced diabetic mice [14]. Results have proven that catalpol was able to reverse mitochondrial dysfunction and increase muscle mitochondrial function and biogenesis, in part by means of the mRNA level upregulation of the peroxisome proliferator-activated receptor gamma co-activator 1 (PGC1α), which is seen especially in body tissues that present a metabolism highly oxidative (heart, skeletal muscle, kidney, brown fat, brain and liver), and shares the responsibility to regulate mitochondrial biogenesis. HFD/STZ-induced muscle mitochondrial dysfunction was shown to be avoided by the interaction between PGC1α and co-activating transcription factors (for example, nuclear respiratory factors, thyroid hormones, α and γ glucocorticoid and estrogen-related receptors), thus elucidating the PGC1α protective role in mitochondria [14]. Additionally, they have been shown to lower circulating total cholesterol and triglyceride levels and fasting blood glucose in a dose-dependent manner, without any body weight variation [14].

Furthermore, it was found that PGC1α contributed to efficiently raise ATP produced in mitochondria, reverse the low potential of the mitochondrial membrane and mtDNA copy number, relieve mitochondrial ultrastructure and increase PGC1α mRNA levels, all by a dose-dependent upregulation. Thus, when catalpol acts as a stimulus for PGC1α-regulated mitochondrial biogenesis, it is capable of lowering the FBG levels, improving mitochondrial function, decreasing oxidative stress and reducing insulin resistance, thus functioning as a good novel approach in T2DM treatment and MS’ insulin resistance management [14,37].

### 4.4. Apigenin-6-C-2”-O-α-L-rhamnopyranosyl)-β-L-fucopyranoside

Apigenin-6-C-2”-O-α-L-rhamnopyranosyl)-β-L-fucopyranoside is a compound isolated from the leaf extract of *Averrhoa carambola* L., which belongs to the Oxalidaceae family, believed to be implicated in stimulating in vivo ^14^C-glucose uptake [82]. As a flavonoid-enriched fraction of the plant, leaves of *A. carambola* demonstrated an efficient reduction of glycemia by means of insulin secretion stimulation and also by the enhancement of glucose-dependent insulin secretion in diabetic rats [82]. Furthermore, this flavonoid-stimulated glucose uptake in rat soleus muscle was driven by the well-known insulin signal transduction mechanism. It is important to note that this effect was nullified when wortmannin (a phosphatidylinositol 3 kinase (PI3K) inhibitor), RO318220 (a protein kinase C inhibitor), mitogen-activated protein kinase (MEK) inhibitor, cycloheximide (a protein synthesis inhibitor) or colchicine (a microtubule-depolymerizing agent) was administered [82]. It was also shown that apigenin-6-C-2”-O-α-L-rhamnopyranosyl)-β-L-fucopyranoside and insulin share no synergistic effect on glucose uptake [30]. This suggests that flavonoids may have a double function, either as an insulin-secretagogue or as an insulin-mimetic agent, and thus considered a new approach in diabetes treatment [82].

### 4.5. Sophora japonica L.

Seed, flower and pericarp of *Sophora japonica* L., from the family Lenguminosea, all contain a great variety of antilipogenic flavonoids, including kaempferol, quercetin and genistein, reported to have anti-lipogenic effects [83]. Among the several polyphenolic compounds extracted from raw products which decrease inflammatory and adipogenic activity in mice, quercetin, found in fruits and vegetables, is responsible for inhibiting glucose uptake and increase lipolysis in adipocytes, while genistein, abundant in soybeans, can decrease food intake, body weight and fat mass [83,84]. Combining together quercetin, genistein and also resveratrol, lipid accumulation significantly decreased in adipocyte cell lines [83,85].

Diets with fruits of *Sophora japonica* L. were found to prevent body weight gain in high-fat diet–induced obesity, decrease body weight gain, reduce serum and hepatic triglyceride, serum total and HDL-CH and decrease large adipocytes and increase small adipocytes in number [83]. These antiobesity effects may be due to the anti-adipogenic compound content, namely, flavonoids [83].

Since obesity is a major risk factor to the development of insulin resistance and T2DM, preventing obesity using mature fruits from *S. japonica* L. as a phytochemical approach makes it possible to control body weight and metabolic diseases with a strict relation to obesity and thus managing both MS and T2DM [83].

### 4.6. Water-Soluble Chitosan

Chitosans belong to a class of natural polymers that are soluble derivatives of chitin, an N-acetyl glucosamine polymer and a polysaccharide from the most commonly occurring ones in nature, mainly found in clams, shellfish krill, squid, oysters, fungi and insects. As chitin is water-insoluble, it is converted to more soluble and lower molecular weight chitosan, a linear copolymer of b-(1,4)-poly-2-amino-2-deoxy-D-glucose and b-(1,4)-poly-2-acetamido-2-deoxy- -D-glucose, by deacetylation and partial hydrolysis reactions [86].

From the great variety of applications, such as those in mining, water treatment, paper industry, biotechnology, food and cosmetic industries and agriculture, soluble chitosans and their derivatives have been increasingly applied in biology and medicine [87,88,89,90,91,92,93,94,95,96,97,98,99]. Sustaining this worldwide application is the fact that chitosan and derivatives seem to be readily absorbed by enterocytes, have low toxicity to eukaryotes, immune system stimulation and hypocholesterolemic and anti-tumor activities [38]. Although solubility is the best property of chitosans, it is highly dependent on molecular weight, residual acetylation degree and chemical modifications [86].

The effect of these water-soluble chitosans on indices of oxidative stress in normal volunteers was reported for the first time in 2009 [86]. The authors demonstrated that chitosan treatment led to a decrease in plasma glucose and HDL-CH levels and atherogenic index [39]. A lowering effect on the oxidized to reduced albumin ratio and an increase in total plasma antioxidant activity (TPA) was also reported [37]. Since the oxidized albumin ratio represents a potentially useful marker of oxidative stress, some in vitro studies were carried out showing a significant decrease in albumin carbonyls and hydroperoxides in a time-dependent fashion, and also the reduction of two stable radicals in a dose- and time-dependent manner, after chitosan administration [86].

These results allow the conclusion that by lowering oxidative stress indices either in vitro or in vivo, chitosan acts as a direct antioxidant agent, which may be an additional benefit to plasma carbohydrate lowering and HDL level increase [86]. Furthermore, it can also inhibit serum albumin oxidation, for example, in diabetic patients who undergo hemodialysis, leading to a reduction of oxidative stress associated with uremia [86].

Chitosans may therefore be an alternative approach in the future of metabolic syndrome and T2DM management, as potent antioxidant agents [86].

### 4.7. Ginseng

Ginseng is one of the most widely used natural compounds. Ginseng root has been used for over 2000 years due to its health-promoting properties. Over the last few years, it has persistently been placed on the top ten of selling herbs in the United States [100,101].

The most commonly used Ginseng species are *Panax ginseng* (Asian ginseng) and *Panax quinquefolius* (American ginseng) [101,102]. In addition, ginseng derivatives may vary in chemical composition depending on the chosen plant extract, root age, growth location, harvest season and drying method. The ginseng species are all made up of ginsenosides, polysaccharides, peptides, polyacetylenic alcohol, and fatty acids [101,102].

Ginseng actions associated with the pharmacological targets are mainly due to ginsenosides, which belong to the steroidal saponin family [103]. Ginsenosides demonstrate an ability to target a myriad of tissues, producing an array of pharmacological responses although their exact mechanism in not well-known [103]. Different hypotheses regarding ginseng’s mechanisms of action have been proposed: (1) ginseng may slow food digestion by a decrease in carbohydrate absorption rate into the portal circulation in liver [100,101]; (2) ginseng can either modify nitric oxide (NO)-mediated glucose transport or contribute to the modulation of the insulin secreted by NO mediation [100,101] and (3) ginseng may interfere in the inflammatory pathway in both insulin-dependent and insulin-independent manners [62].

There are very few known ginseng adverse effects and the most common ones are nervousness and excitation, which may be reduced by prolonged use or dosage reduction [100,101].

### 4.8. Momordica charantia (Bitter Melon)

*Momordica charantia* or bitter melon is widely cultivated in Asia, Africa, and South America [104,105]. The hypoglycemic action of the fresh juice or unripe fruit was reported after in vivo studies in animal experimental models and also human clinical trials. Bitter melon preparations orally administered have shown promising results in clinical trials involving T2DM patients [106].

### 4.9. Trigonella foenum graecum (Fenugreek)

*Trigonella foenum graecum* has long been used for its anti-diabetic, anti-oxidant, and anti-inflammatory properties [107]. The active compound is found in the non-fatted portion of the seed, which also contains saponins, flavonoids, lipids, vitamins and minerals. Moreover, literature reports the liver protective, obesity combating, hypoglycemic, anti-diabetic and cholesterol lowering properties of the seed [108,109]. In particular, the glucose- and lipid- lowering effects were also noticed in human studies [110]. At least 50% of the seed is composed of fiber which may be viewed as another possible mechanism of fenugreek’s action in T2DM patients, since it promoted a great reduction of both fasting plasma and post-prandial blood glucose levels [110]. The antidiabetic effects of the seeds are attributed to galactomannan which decreases insulin resistance and glucose resorption from the gastrointestinal tract; 4-hydroxyisoleucin (4-OH-Ile) which increases insulin secretion, diosgenin and trigonelline due improving β-cell regeneration [111].

### 4.10. Gymnema sylvestre (Gurmar)

*Gymnema sylvestre* is a tropical plant native to the Indian forests and also a well-known Ayurvedic anti-sweet plant used in T2DM treatment [70]. It is an important fact that *Gymnema sylvestre* varies in its chemical composition depending on the geography: species from Vietnam are not equal to those in India. In 3T3-L1 adipocyte cells, gymnemosides produce a stimulating effect on 2-NBDG uptake, thus being promising as lead molecules for novel anti-diabetic therapies [70].

*Gymnema sylvestre* is known as a “sugar blocker” [112]. In a T2DM clinical study, *Gymnema sylvestre* extract was administered daily along with oral antidiabetic drugs [113]. All patients showed an improvement on glycemic control and, when combining both therapies, it was possible to considerably reduce oral hypoglycemic drug dosage or even discontinue oral medication and maintain glycemic control with the *Gymnema* extract alone, as it has succeeded with five of these patients.

### 4.11. Allium cepa and Allium sativum

Both *Allium cepa* (onions) [114] and *Allium sativum* (garlic) [115] have shown a good performance as blood sugar lowering agents in several studies [116,117,118]. Volatile oils present in either raw onion or garlic cloves revealed a fasting glucose level lowering effect in diabetic animal models and humans. Sulfur-containing compounds—allyl propyl disulfide (APDS) in onions and diallyl disulfide (allicin) in garlic—seem to be the active constituents of these products. Since organosulfur compounds (OSCs) from plant extracts have shown antioxidant, blood glucose lowering, anti-inflammatory and immunomodulatory actions, they may be considered as cardioprotective in T2DM. Garlic OSCs have the interesting feature of gasotransmitter H2S production and should be incorporated in the dietary regimen of type 2 diabetic individuals [52,119]. These active ingredients are able to reduce glycemia by competition against insulin for liver insulin-inactivating sites, thus producing high levels of free insulin [52]. Sulfur compounds such as *S*-methylcysteine and flavonoids (e.g., quercetin) are the main responsible compounds for the glucose-lowering ability of *Allium cepa*. and also participate in the reduction of serum lipids, oxidative stress and lipid peroxidation, in parallel to an increased antioxidant activity and insulin secretion [119].

### 4.12. Pterocarpus marsupium and other Epicatechin-Containing Plants 

From the bark of *Pterocarpus marsupium* plants, used long ago by Indian people to manage diabetes, the flavonoid epicatechin may be extracted [120]. This compound acts in beta-cell damage prevention, as shown in rats. Additionally, studies performed in diabetic animal models with either epicatechin or a crude alcohol extract from P. marsupium have revealed their ability to regenerate pancreatic beta-cells with a preserved function [121]. Among the components of P. marsupium, epicatechin and catechin, which belong to flavan-3-ols family, are composed of glycosides and esters that may be responsible for their anti-diabetic action [122]. Similarly, both Camellia sinensis (green tea polyphenols) and Acacia cate-chu (Burma cutch) may be considered as flavan-3-ols enriched compounds. Cathecins such as epigallocathecin gallate have been proposed as new actives in nanoformulations for the management of diabetic retinopathy [123,124,125,126]. 

### 4.13. Vaccinium myrtillus (Bilberry)

The shrub *Vaccinium myrtillus*, also known as bilberry or European blueberry, typically grows in Europe and its leaves are increasingly used for diabetes treatment prior to marketed insulin availability [127]. However, the fruit has also beneficial properties attributed to the presence of Anthocyanoside, a compound that reduces fasting glucose, serum cholesterol and HbA1C in T2DM patients [128].

Several bioactive components (e.g., vitamins, phenolic acid and chlorogenic acid) which are increasingly incorporated in blueberry fruits, may contribute to a protective effect by (i) oxidative stress inhibition, (2) blood glucose metabolism control, (3) lipid profile improvement and (4) inflammatory cytokine lowering activity [73,128].

### 4.14. Atriplex halimus (Salt Bush)

*Atriplex halimus*, commonly known as salt bush, is a plant endemic to Israel; its aqueous leaf extract has demonstrated to reduce blood and liver glucose levels [129]. In vivo studies performed with diabetic rats (sand rats) revealed the T2DM onset in the absence of the *A. hamilus* [130].

### 4.15. Aloe vera

*Aloe vera* has long ago been recognized as a traditional therapy for diabetes management [131]. Orally administered *aloe vera* juice produced from the plant leaves (*aloe vera* gel) has shown promising results in reducing fasting glycemia and triglycerides in T2DM patients, either alone or combined with any conventional antidiabetic drug [132]. *Aloe vera* contains phytosterols that exhibit long-term glycemic control, being active against diabetes type II [133].

In addition, clinical trials undertaken with Aloe gel show that it may be a safe anti-hyperglycemic and anti-hypercholesterolemic agent for T2DM patients presenting with hyperlipidemia [134,135].

## 5. Conclusions

As diabetes mellitus is a metabolic disorder related to complex disturbances in carbohydrate, protein and fat metabolisms, interest remains to discover innovative and highly-effective glucose-lowering agents, with minimal adverse effects and low toxicity. Currently available pharmacological options discussed in this review include sulphonylureas, thiazolidinediones, α-glucosidase inhibitors, sodium-glucose co-transporter type 2 inhibitors and dual SGLT1/SGLT2 agonists, glucagon-like peptide-1 amide (GLP-1) receptor agonists, dipeptidyl-peptidase-4 inhibitors, bile acid sequestrants, dopamine type 2 receptor agonists, amylin mimetics, mangiferin-berberine salt, β-tigogenin cellobioside, incretin-based therapies, TGR 5 agonists, β-cell acting compounds, small-molecule insulin releasers, glucokinase activators, fatty Acid Receptor Agonists, imeglimin, adipokine-based treatments, selective peroxisome proliferator-activated receptor (PPAR) modulators, 11β-hydroxysteroid dehydrogenase 1 inhibitors, antiobesity drugs, 55P0110, antiosteoporotic trace minerals: Silicon and strontium, hybrid Molecules and Hybrid natural products, bis(α-furancarboxylato)oxovanadium (IV), LR Compounds: LR-9 and LR-74, insulinomimetic zinc (II) complexes with natural products, 4-(2,2-dimethyl-L-oxopropyl)benzoic acid, boronated nucleosides and nucleotides. Natural compounds such as diplacone and mimulone, ethanolic extract of *Semecarpus anacardium* (Linn.) bark, catalpol, apigenin-6-C-2”-O-α-L-rhamnopyranosyl)-β-L-fucopyranoside, *Sophora japonica* L., water-soluble chitosan, *ginseng, Momordica charantia, Trigonella foenum graecum, Gymnema sylvestre, Allium cepa* and *Allium sativum, Pterocarpus marsupium* and other *Epicatechin-containing plants*, *Vaccinium myrtillus, Atriplex halimus* and *aloe vera*, have also been considered as potential therapeutic approaches. Alongside drug research, a meticulous selection of the raw materials, animal models for in vivo experiments and also proper routes to administrate the testing compounds must be ensured.

## Figures and Tables

**Table 1:** Main targets of the hypoglycemic drugs currently approved for the treatment of diabetes mellitus (Adapted from [33]).

Drug Targets	Hyperglycemic Effect Observed with:
Insulin secretion	↗ Incretins ↗ Meglitinides ↗ Sulfonylureas
Glucagon secretion	↓ incretins ↓ amylin
Gastrointestinal tract	Incretins Alpha-glucosidase inhibitors Amylin Sequestrant of bile salt
Hepatic glucose output	↓ Metformin ↓ Thiazolidinediones
Lipotoxicity	Thiazolidinediones
Control of appetite	Incretins Amylin
Neurotransmitter Dysfunction	Bromocriptine
Glucose reabsorption	↓ Gliflozins inhibitors
Glucose uptake and use	↗ Metformin ↗ Thiazolidinediones
